# Changes in the concentrations of trimethylamine N-oxide (TMAO) and its precursors in patients with amyotrophic lateral sclerosis

**DOI:** 10.1038/s41598-020-72184-3

**Published:** 2020-09-16

**Authors:** Lu Chen, Yong Chen, Mingming Zhao, Lemin Zheng, Dongsheng Fan

**Affiliations:** 1 Department of Neurology, Peking University Third Hospital, 49 North Garden Road, Haidian District, Beijing, China; 2 School of Basic Medical Sciences, Institute of Cardiovascular Sciences and Institute of Systems Biomedicine, Peking University Health Science Center, Beijing, China; 3 Key Laboratory of Molecular Cardiovascular Sciences of Ministry of Education, Peking University Health Science Center, Beijing, China; 4 Key Laboratory for Neuroscience, National Health Commission/Ministry of Education, Peking University, Beijing, China

**Keywords:** Diseases, Medical research, Neurology

## Abstract

To compare the plasma concentrations of trimethylamine N-oxide (TMAO) and its precursors in amyotrophic lateral sclerosis (ALS) patients, their spouses and healthy controls and to find associations between gut microbiota metabolites and ALS. ALS patients were recruited at Peking University Third Hospital from January 2015 to December 2018. Information was collected from their spouses at the same time. Age and gender matched healthy controls were recruited from individuals who visited the physical examination center for health checkups. Blood samples were collected after at least 4 h of fasting. Concentrations of the metabolites were quantified using stable isotope dilution liquid chromatography–tandem mass spectrometry. Group differences were analyzed using parametric and nonparametric tests, as appropriate. In this study, 160 patients with ALS were recruited. In these patients, 63 were compared with their spouses, 148 were compared with age and gender matched controls, and 60 were compared with both their spouses and heathy controls in the same time. The carnitine concentration was significantly higher in patients than in their spouses, while there were no significant differences in the concentrations of other metabolites. The carnitine and betaine concentrations were higher, while the choline, TMAO and butyrobetaine concentrations were lower in ALS than in healthy controls. The concentrations of the metabolites in the spouses were more similar to the ALS patients rather than to the healthy controls. In the ALS group, the plasma concentrations of carnitine, betaine, choline and TMAO were inversely related to the severity of upper motor neuron impairment. The TMAO metabolic pathway of the gut microbiota is disturbed in both ALS patients and their spouses, which might suggest that the changes in the gut microbiota occurred before disease onset. The negative correlations between the involvement of UMNs and the concentrations of the metabolites might suggest that the inhibition of this metabolic pathway might lead to a better prognosis in ALS patients.

## Introduction

Amyotrophic lateral sclerosis (ALS) is a fatal neurodegenerative disease that involves upper and lower motor neurons^1–8^. The etiology of ALS is still unknown, and the average survival time is 3–5 years^3,6–10^. It is believed that both genes and the environment play roles in ALS^8,11–13^. It is important to study the pathogenesis and prognostic factors of ALS to improve the quality of life and prolong the survival of patients^7^.

Recently, with advancements in the understanding of the gut microbiota, increasing attention has been paid to the associations between the gut microbiota and neurodegenerative diseases, such as Alzheimer’s disease and Parkinson’s disease^14–16^. With regard to ALS, as early as 2005, researchers hypothesized that the neurotoxins produced by clostridial species in the gut might be the cause of ALS in susceptible individuals^17^. In 2015, Wu et al.^18^ confirmed that the intestinal microbiota in transgenic mice that carried a human SOD1 gene with a G93A mutation (SOD1^G93A^ mice) shifted before the onset of ALS symptoms compared with wild-type mice. A recently published study also confirmed that the gut microbiota was significantly changed in ALS SOD1^G93A^ mouse models compared with controls and that the disease was exacerbated under germ-free or wide-spectrum antibiotic treatment conditions^19^.

TMAO is the oxidation product of trimethylamine (TMA) and is an important metabolite of gut microbiota^20^. The main sources of TMAO are choline and carnitine from foods^20,21^. Although L-carnitine and TMAO are believed to be causes of atherosclerosis^20,21^, Kira et al. confirmed that the oral administration of L-carnitine may delay the onset and progression of ALS and extend the life span of SOD1^G93A^ mice^13,22^. Besides, a randomized double-blind placebo-controlled trial of acetyl-L-carnitine for ALS conducted by Beghi et al.^23^ confirmed that it was effective, well-tolerated and safe in ALS. The effectiveness of carnitine treatment suggested that TMAO and its related metabolites may play important roles in the onset and progression of ALS.

Studying the relationship between TMAO and ALS may help us further understand the disease. In this article, we compared the concentrations of TMAO and its precursors in the plasma of patients with ALS, their spouses and age and gender matched healthy controls, and tried to find associations between the metabolites of the gut microbiota and ALS.

## Methods

This case–control study was approved by the institutional ethics committee of Peking University Third Hospital (PUTH; IRB00006761). All the methods were carried out in accordance with relevant guidelines and regulations.

Since the concentrations of TMAO, betaine, choline and butyrobetaine in the plasma were very closely related to the food^20,21,24,25^, comparison of the levels of these metabolites between ALS patients and their spouses might minimize the influence of food, because most ALS patients and their spouses live together and eat similar foods. Besides, the concentrations of these metabolites were also compared between patients with ALS and age and gender matched healthy controls.

### Participants

The ALS group was composed of adult participants who met the following criteria: between 18 and 75 years old; probable supported by laboratory findings, probable, or definite ALS diagnosis according to the revised El Escorial criteria; normal function of the bulbar muscles; and no difficulty swallowing. Women of childbearing age were excluded if pregnant or breastfeeding. The exclusion criteria included abnormal findings on electrodiagnostic, neurophysiologic, neuroimaging or clinical laboratory studies that could not be explained by ALS, the presence of dementia or psychiatric disorders; the presence of gastrointestinal disorders or the performance of gastrointestinal surgery that might affect gastrointestinal absorption; severe diseases of the heart, liver, kidney or other organs; and treatment with antibiotics, L-carnitine or intestinal flora regulation within 3 months before enrollment.

For both the group of spouses of patients with ALS and the healthy control group, eligible participants were adults between 18 and 75 years old who had normal function of the bulbar muscles and could eat normally. Exclusion criteria included pregnancy or breastfeeding, dementia or psychiatric disorders, a history of gastrointestinal disorders or gastrointestinal surgery, and severe diseases of other organs. The participants in healthy control group were matched with patients in the ALS case group by age and gender.

All the participants volunteered to participate in the study, and informed consent was signed by the participants or their authorized relatives.

### Information collection

Patients were recruited from January 2015 to December 2018, and each patient was given a follow-up evaluation by telephone every 3 months. For all patients who agreed to participate in the study, baseline demographic information, clinical data and blood samples were collected during the patient’s first visit to PUTH, and further information was collected during follow-up evaluations. Patients were diagnosed and classified according to the revised El Escorial criteria^26^. All patients were examined by two board-certified neurologists from the study group who were experienced in the diagnosis of motor neuron diseases. If the diagnoses or disease categories determined by the neurologists differed, a third more experienced neurologist examined the patient to make the final determination. All the demographic and clinical information of the participants was recorded on the case report forms (CRFs), and the CRFs were kept on paper and as Epidata forms. Information and blood samples from the spouses of the patients with ALS was also collected when the patients visited PUTH. The healthy controls were recruited from the individuals who came to the physical examination center for health checkups. In this study, the gender and age were matched between patients with ALS and the healthy controls.

### Measurements of the concentrations of choline, carnitine, betaine and TMAO in the plasma

Blood samples were collected from all the participants after fasting for at least 4 h. All the samples were sent for testing at the Peking University Institute of Cardiovascular Sciences. The concentrations of TMAO and its precursors were quantified using stable isotope dilution liquid chromatography–tandem mass spectrometry, as described previously with little modification (10.1016/j.jchromb.2016.09.026; 10.1016/j.freeradbiomed.2018.01.007; 10.1111/acel.12768). Briefly, 20 μl of plasma was aliquoted into a 1.5 ml tube and mixed with 80 μl of 10 μM internal standard composed of d9-TMAO, d11-betaine, d9-carnitine, d9-butyrobetaine and d9-choline in methanol. The protein in the samples was precipitated, and the supernatant was recovered following centrifugation at 20,000 g at 4 °C for 10 min. Supernatants were analyzed by injection into a silica column (2.0 * 150 mm, Luna 5u Silica 100A; Cat. No. 00F-4274-B0, Phenomenex, Torrance, CA) at a flow rate of 0.4 ml/min using an LC-20AD Shimadazu pump system, a SIL-20AXR autosampler interfaced with an API 5500Q-TRAP mass spectrometer (AB SCIEX, Framingham, MA). The LC gradient condition and targeted MS instrument parameters were presented in Table S1. Analytes were monitored using electrospray ionization in positive-ion mode with multiple reaction monitoring (MRM) of precursor and characteristic product-ion transitions. The detailed mass spectrometric parameters for targeted compounds were listed in Table S2. Standard curves were generated, and the standard curves were acceptable when the coefficient of determination (R^2^) reached 0.999. And the accuracy of analytes (TMAO, Betaine, Choline and Carnitine) concentration was calculated and listed in Table S3.

### Data analysis

All data were analyzed with SPSS V16.0 software (SPSS, Chicago, Illinois, USA), and significance was set at 5%. Normality tests were performed for all continuous variables. Since data of the concentrations of the metabolites were not normally distributed, medians rather than averages were used in the analysis. Parametric tests [one-way analysis of variance (ANOVA) or Student’s *t* test] or nonparametric tests (χ^2^ test, Fisher’s exact test, Kruskal–Wallis one-way ANOVA by ranks, or Mann–Whitney U test) were used to compare differences between subgroups, as appropriate. Spearman rank correlation was used to analyze the correlations between the level of metabolites in the serum and the clinical features of patients with ALS.

### Definitions

In the database, patients with ‘familial ALS’ were defined as patients with at least one family member with ALS. The ‘use of riluzole’ was defined as treatment with riluzole (50 mg) twice a day for longer than 2 weeks. ‘Spouses of the patients’ were defined as the people who married to the patients and lived together.

In this study, the involvements of the upper and lower motor neurons were evaluated in the four regions of the spine: bulbar, cervical, thoracic and lumbosacral regions. The involvements of upper and lower motor neurons in the four regions were scored separately and any involvement was scored as one point. Then the scores for upper and lower motor neuron involvement were separately added up for a total of four points. The relative severity of the upper and lower motor neuron impairment was represented by the ratio of the upper motor neuron (UMN) involvement score to the lower motor neuron (LMN) involvement score (U:L ratio). A higher U:L ratio suggested that the impairment of the UMNs was relatively more severe.

## Results

In this study, 160 patients with ALS were recruited. In these patients, 148 were compared with age and gender matched healthy controls, 63 were compared with their spouses. Then in the 63 patients with ALS who had the data of their spouses, 60 were compared with both their spouses and heathy controls in the same time. The demographic features and clinical characteristics of the patients with ALS who were recruited in the study were shown in Table 1.

**Table 1:** Demographic and clinical characteristics of the patients with ALS.

	All patients with ALS	Patients compared with their spouses	Patients compared with healthy controls
Total, n	160	63	148
Age, year [mean (95% CI)]	53.98 (52.50–55.46)	51.93 (49.87–54.00)	53.76 (52.19–55.34)
Male to female ratio	1.58:1	1.73:1	1.51:1
Diagnostic delay, day [median (IQR)]	385.5 (462)	401.0 (349)	388.5 (457.75)
FRS-R score [median (IQR)]	41.0 (9)	39.0 (10)	41.0 (9)
**Phenotype, n (%)**			
Limb-onset ALS	98 (61.3)	38 (60.3)	91 (61.5)
Bulbar-onset ALS	27 (16.9)	11 (17.5)	26 (17.6)
FAS	24 (15.0)	9 (14.3)	20 (13.5)
PMA	4 (2.5)	1 (1.6)	4 (2.7)
Familial ALS	7 (4.4)	4 (6.3)	7 (4.7)
**Category, n (%)**			
Definite	65 (40.6)	26 (41.3)	60 (40.5)
Probable	42 (26.3)	17 (27.0)	39 (26.4)
Probable supported by laboratory findings	24 (15.0)	8 (12.7)	22 (14.9)
Possible	27 (16.9)	11 (17.5)	25 (16.9)
Pure LMN impairment	2 (1.3)	1 (1.6)	2 (1.4)
Riluzole, n (%)	122 (76.3)	46 (73.0)	112 (75.7)
Smoking, n (%)	53 (33.1)	24 (38.1)	49 (33.1)
Alcohol abuse, n (%)	44 (37.5)	22 (34.9)	39 (26.4)
History of contact with pesticides, n (%)	28 (17.5)	10 (15.9)	24 (17.0)

*ALS* amyotrophic lateral sclerosis, *FAS* flail arm syndrome, *PMA* progressive muscular atrophy, *LMN* lower motor neuron.

### Clinical features and analysis of the ALS patients

Among the 160 patients with ALS, the mean age was 53.98 years old (95% confidence interval (CI), 52.50–55.46), and 98 patients were males (M:F ratio was 1.58:1). Among these patients, 98 (61.30%) had limb-onset ALS, 27 (16.90%) had bulbar-onset ALS, 24 (15.00%) had flail arm syndrome (FAS), 4 (2.50%) had progressive muscular atrophy (PMA) and 7 (4.40%) had familial ALS. According to the revised El Escorial criteria, the diagnostic categories at the first visit were definite for 65 (40.60%) patients, probable for 42 (26.30%), probable supported by laboratory findings for 24 (15.00%), possible for 27 (16.90%), and uncategorized for 2 patients (1.25%) with only LMN impairment. The percentage of patients who used riluzole was 76.3% (122 patients). In total, 33.1% of the patients had a history of smoking, and 37.5% of the patients had a history of long-term alcohol consumption. A total of 17.5% of the patients had used pesticides. For these patients, the median diagnostic delay was 385.5 days [Interquartile range (IQR), 462] and the median revised ALS functional rating scale (ALSFRS-R) score was 41.0 (IQR, 9) (Table 1).

In the 160 patients, the median concentrations of the metabolites in the plasma were 47.10 μmol/L (IQR 12.90) for carnitine, 45.67 μmol/L (IQR 22.13) for betaine, 5.18 μmol/L (IQR 2.31) for choline, 1.80 μmol/L (IQR 1.76) for TMAO and 1.02 μmol/L (IQR 0.49) for butyrobetaine. Males have higher concentrations of choline (*p* < 0.0005) and butyrobetaine (*p* = 0.002), while there was no significant difference in the concentrations of carnitine (*p* = 0.106), betaine (*p* = 0.149) and TMAO (*p* = 0.220) between males and females. In the Spearman analysis, the concentrations of carnitine (*p* = 0.048), betaine (*p* < 0.0005) and choline (*p* < 0.0005) were positively related with age, while the concentrations of butyrobetaine (*p* = 0.668) and TMAO (*p* = 0.061) were not relevant with age. Besides, the concentrations of carnitine (*p* = 0.021), betaine (*p* = 0.034), choline (*p* = 0.028) and TMAO (*p* = 0.049) in the plasma were all inversely related to the relative severity of UMN impairment. The concentrations of the metabolites were not correlated with the diagnostic delay, FRS-R scores or the diagnostic categories at the first visit.

### Comparison between the ALS group and the spouse group

There were 63 patients in the ALS group, and 40 patients were males. The male to female (M:F) ratio was 1.73:1. The detailed clinical features of patients were shown in Table 1. Correspondingly, there were 63 spouses of the patients in the spouse group. The mean age of the ALS group [51.93 years old (95% CI, 49.87–54.00)] was comparable with that of the spouse group [50.26 years old (95% CI 46.81–53.72)] (*p* = 0.97). The concentration of carnitine was significantly higher in the patient group [46.77 μmol/L, interquartile range (IQR) 13.84] than in the spouse group (38.58 μmol/L, IQR 9.52) (*p* < 0.0005), while there were no significant differences in the concentrations of choline, betaine, butyrobetaine and TMAO. (Table 2).

**Table 2:** Comparisons of the concentrations of TMAO and its precursors in the plasma between patients and other groups.

Metabolites, μmol/L [Median (IQR)]	Patients with ALS compared with their spouses			Patients with ALS compared with healthy controls		
	Patients	Spouses	*p*	Patients	Controls	*p*
Carnitine	47.84 (13.84)	38.58 (9.52)	< 0.0005	46.86 (12.89)	44.47 (11.37)	0.023
Choline	5.04 (2.33)	4.73 (1.81)	0.104	5.03 (2.25)	5.64 (2.55)	0.002
Betaine	40.90 (15.19)	40.10 (15.66)	0.971	45.16 (20.79)	36.22 (12.00)	< 0.0005
Butyrobetaine	0.82 (0.53)	0.75 (0.48)	0.529	0.99 (0.53)	1.50 (0.74)	< 0.0005
TMAO	1.35 (1.55)	1.09 (0.86)	0.195	1.76 (1.76)	2.29 (1.70)	0.001

*ALS* amyotrophic lateral sclerosis, *TMAO* trimethylamine N-oxide.

### Comparison between the ALS group and the age and gender matched healthy control group

There were 148 participants in each group, and the M:F ratio was 1.51:1. The mean age was 53.76 years old (95% CI 52.19–55.34) in the ALS group and 53.78 years old (95% CI 52.17–55.38) in the healthy control group. Age (*p* = 0.991) and sex (*p* = 1) were matched between the patient group and the healthy control group. The detailed clinical features of patients with ALS were shown in Table 1. The concentrations of carnitine (*p* = 0.023) and betaine (*p* < 0.0005) were higher, while the concentrations of choline (*p* = 0.002), TMAO (*p* = 0.001) and butyrobetaine (*p* < 0.0005) were lower in the ALS group than in the healthy control group. All the detailed results are shown in Table 2.

### Comparison between the ALS patients, their spouses and the age and gender matched healthy controls

The results above showed that the concentrations of the metabolites in the plasma of the spouses of patients with ALS were more similar to those of patients with ALS rather than those of the healthy controls, so a direct comparison between the ALS patients, their spouses and the age and gender matched healthy controls was conducted. There were 60 participants in each group, and there were 37 males in the group of patients with ALS and the healthy control group, and 23 males in the spouse group. The mean age was 51.87 years old (95% CI 49.69–54.04) in the ALS group, 52.18 years old (95% CI 49.70–54.66) in the spouse group and 53.23 years old (95% CI 50.89–55.57) in the healthy control group. The age was comparable between the three groups (*p* = 0.732). The concentration of carnitine was significantly higher in the ALS patient group than in the spouse group (*p* < 0.0005), while there were no significant differences in the concentrations of choline, betaine, butyrobetaine and TMAO. The concentrations of betaine (*p* = 0.041) were higher, while the concentrations of choline (*p* = 0.031), TMAO (*p* < 0.0005) and butyrobetaine (*p* < 0.0005) were lower in the ALS group than in the healthy control group. The concentrations of carnitine (*p* < 0.0005), betaine (*p* = 0.034), choline (*p* < 0.0005), butyrobetaine (*p* < 0.0005) and TMAO (*p* < 0.0005) were all significantly different between the spouses of patients and the healthy controls. All the detailed results are shown in Table 3.

**Table 3:** Comparisons of the concentrations of TMAO and its precursors in the plasma between the ALS patients, their spouses and healthy controls.

Metabolites, μmol/L [Median (IQR)]	Participants			*p*
	Patients	Spouses	Controls	
Carnitine	46.73 (15.16)	38.80 (9.30)	44.37 (10.84)	< 0.0005
Choline	5.03 (2.26)	4.74 (1.76)	5.57 (2.51)	0.053
Betaine	40.43 (14.47)	40.23 (16.23)	35.82 (11.12)	0.002
Butyrobetaine	0.79 (0.52)	0.73 (0.47)	1.52 (0.83)	< 0.0005
TMAO	1.39 (1.51)	1.05 (0.82)	2.29 (2.09)	< 0.0005

*ALS* amyotrophic lateral sclerosis, *TMAO* trimethylamine N-oxide.

## Discussion

This study focused on the relationship between the metabolites of the gut microbiome and ALS. We have several findings. First, the concentrations of TMAO and its precursors in both patients with ALS and their spouses were significantly different from the age and gender matched healthy controls. Second, the concentrations of the metabolites in the plasma of patients with ALS were inversely correlated with the impairment of UMNs.

As an important metabolite of the gut microbiome, TMAO has been found to be associated with several diseases, such as atherosclerosis, inflammatory bowel disease, chronic kidney disease and autism spectrum disorders^27–34^. Relationships between the gut microbiota and neurodegenerative diseases have been reported^14,15,35,36^, and changes in the gut microbiota and the associated metabolites have been found in many previous studies of ALS^19,36,37^. Sun et al.^38^ reported that the use of antibiotics might be associated with a higher subsequent risk of ALS, which suggested that the disturbances of the gut microbiota were a potential cause of this neurodegenerative disease. However, the role of TMAO in ALS has not been investigated before. In this article, we report several primary findings.

First, compared with their spouses, patients with ALS had elevated plasma concentrations of carnitine, while compared with healthy controls, ALS patients had significantly different concentrations of several metabolites. Since the concentrations of TMAO and its precursors were relevant with age and gender, the matched age and sex between patients and controls avoided the influence of these two factors in the analysis. Previous studies have confirmed that carnitine can be biosynthesized endogenously and be absorbed from the gut, while TMAO can only be produced by gut microbiota metabolism^20,21,39^. Since choline and butyrobetaine are produced through the gut microbe-mediated metabolism of dietary phosphatidylcholine and carnitine, respectively, the decreased concentrations of these two metabolites and the decreased concentration of TMAO in the plasma of patients with ALS compared with healthy individuals suggests that there are disturbances to the absorptive and metabolic functions of the gut microbiota, while the elevated plasma concentration of carnitine might be the result of increased endogenous biosynthesis^20,21,25,36,39^. In addition, as one of the important precursors of TMAO, carnitine is a critical component in the metabolism of long-chain fatty acids in the mitochondria of human cells^23,40,41^. The increased endogenous biosynthesis of carnitine in patients with ALS suggests a state of hypermetabolism and deficits in the metabolism of fatty acids, which have been reported in previous studies^13,42–44^. Defective energy metabolism and abnormal mitochondrial function partly explain the poor prognosis of patients with ALS^42^.

It is quite interesting that the concentrations of TMAO and its precursors in the plasma of the patients’ spouses were much closer to those in the plasma of the patients with ALS than to those in the plasma of the healthy controls. These changes showed that there were some disturbances in the gut microbe-mediated metabolism of choline and carnitine in the spouses of the patients with ALS, although they were all healthy individuals. The changed concentrations of these metabolites in the plasma of the spouses may be evidence that the change in the gut microbiota occurred before the onset of the disease^38^, while the susceptibility to the disease was different between individuals. This finding also supports the opinion that ALS involves gene-environment interactions^13^. However, the reason for the similar changes in the gut microbiota of patients with ALS and their spouses was not identified in this study. Since the concentrations of TMAO and its precursors in the plasma are mainly influenced by food^20,21,24,25^, the similar diet that the patients with ALS and their spouses ate might contribute to this change, although more convincing evidence is needed to support this idea.

Second, in patients with ALS, the concentrations of TMAO and its precursors in the plasma were inversely correlated with the impairment of UMNs. Compared with the data from our previous cohort studies of the same population, the diagnostic delay was relatively shorter in this study^1,45^, which might suggest that patients recruited here were in a relatively earlier stage of the disease and that the concentrations of the metabolites had already changed in this early stage. The concentrations of TMAO and its precursors were independent from the diagnostic delay, FRS-R scores and diagnostic category of the patients, which might suggest that there was little relationship between the stage of the disease and the levels of the metabolites. However, the data obtained in our study showed that the higher the concentration of carnitine in the plasma was, the less the involvement of UMNs. As mentioned above, since carnitine plays an important role in the metabolism of long-chain fatty acids, elevated concentrations of carnitine are a sign of hypermetabolism^42–44^. Several studies have confirmed that hypermetabolism is a factor leading to a poor prognosis in ALS, and less severe hypermetabolism may be an explanation for the better prognosis of patients with UMN-dominant ALS^43,44,46^. In addition, the negative correlations between the involvement of UMNs and the concentrations of TMAO and other metabolites suggests that the inhibition of this metabolic pathway may lead to a better prognosis in patients with ALS.

This study has several limitations. First, different dietary habits between the study participants might have partially accounted for the findings of this study. Second, although the sample size in this study was relatively large, some of the subgroup analyses were still limited by the lack of sufficient statistical power. Third, the potential impacts of other factors, such the use of antibiotics, which have been reported as potential risk or protective factors for ALS, were not analyzed^47^. Last, even though our analyses were based on a causal hypothesis, the findings are only suggestive and cannot determine causality^25^. Future studies that include more information on confounding factors are needed to confirm our findings.

In conclusion, compared with the heathy controls, the TMAO metabolic pathway of the gut microbiota is disturbed in both ALS patients and their spouses, which might suggest that the changes in the gut microbiota occurred before disease onset. In addition, the negative correlations between the involvement of UMNs and the concentrations of TMAO and other metabolites might suggest that the inhibition of this metabolic pathway might lead to a better prognosis in patients with ALS.

## Supplementary information

Supplementary Tables.
