# Supplementary material for: Changes in the concentrations of trimethylamine N-oxide (TMAO) and its precursors in patients with amyotrophic lateral sclerosis
Source: Sci Rep. 2020 Sep 16;10:15198. doi: 10.1038/s41598-020-72184-3 (PMC7495434; doi:10.1038/s41598-020-72184-3)
Supplement: Supplementary file 1 — Supplementary Tables. [file 41598_2020_72184_MOESM1_ESM.docx]

**Changes in the concentrations of trimethylamine N-oxide (TMAO) and its precursors in patients with amyotrophic lateral sclerosis**

Lu Chen^a^, Yong Chen^a^, Mingming Zhao^b,c^, Lemin Zheng^b,c,*^, Dongsheng Fan^a,d,*^

*^a^Department of Neurology, Peking University Third Hospital, Beijing, China*

*^b^School of Basic Medical Sciences, Institute of Cardiovascular Sciences and Institute of Systems Biomedicine, Peking University Health Science Center, Beijing, China*

*^c^Key Laboratory of Molecular Cardiovascular Sciences of Ministry of Education, Peking University Health Science Center, Beijing, China*

*^d^Key Laboratory for Neuroscience, National Health Commission/Ministry of Education, Peking University, Beijing, China*

*These authors contributed equally

Correspondence:

Dongsheng Fan, 49 North Garden Road, Haidian District, Beijing, China. E-mail: dsfan2010@aliyun.com

Supplemental tables

Table S1: The detailed information of LC gradient condition and targeted MS instrument parameters.

| **Methods** | **TMAO** |
| --- | --- |
| Column | Luna 5u Silica 100A, 2.0*150 mm |
| Column Chamber T (℃) | 35 |
| Flow Rate (mL/min) | 0.4 |
| Injection Volume (μL) | 3 |
| Mobile Phase A | H_2_O / propanoic acid = 100 / 0.1 |
| Mobile Phase B | MeOH / acetic acid = 100 / 0.1 |
| Gradient (B %) | 0.50min-5%  4.00min-95%  5.00min-95%  5.50min-5%  5.80min-stop |
| Q TRAP5500 | Positive |
| Spray voltage (kV) | 5.5 |
| Source temperature (℃) | 600 |
| Collision activation parameter | Medium |
| Curtain Gas (psi) | 35 |
| GS1 (psi) | 60 |
| GS2 (psi) | 60 |

Table S2: Mass spectrometric parameters for targeted compounds.

| Analytes | MRM transition | dwell | DP | CE | EP | CXP |
| --- | --- | --- | --- | --- | --- | --- |
| TMAO | 75 → 58 | 100 | 94 | 24.3 | 10 | 11 |
| d9-TMAO | 85 → 66 | 100 | 65 | 28 | 10 | 11 |
| Choline | 104 → 59.8 | 100 | 84 | 22 | 10 | 11 |
| d9-Choline | 113.2 → 68.9 | 100 | 70 | 24.1 | 10 | 11 |
| Betaine | 118 → 59 | 100 | 85 | 38 | 10 | 11 |
| d11-Betaine | 129.1 → 65.9 | 100 | 86 | 37 | 10 | 11 |
| Butyrobetaine | 146.1 → 87 | 100 | 85 | 25.1 | 10 | 11 |
| d9-Butyrobetaine | 155.1 → 87 | 100 | 95 | 24.9 | 10 | 11 |
| Carnitine | 162 → 103 | 100 | 80 | 20.2 | 10 | 11 |
| d9-Carnitine | 171.1 → 102.8 | 100 | 98 | 24.9 | 10 | 11 |

Table S3: The internal standards were spiked in the samples and the accuracy of analyte (TMAO, Betaine, Choline and Carnitine) concentration was calculated.

| Standard concentrations(μM) | TMAO Accuracy (%) |
| --- | --- |
| 0.195 | 94.1 |
| 0.39 | 91.9 |
| 0.78 | 101. |
| 1.56 | 104. |
| 3.125 | 99.2 |
| 6.25 | 104. |
| 12.5 | 97.7 |

| Standard concentrations(μM) | Choline Accuracy (%) |
| --- | --- |
| 1.56 | 95.3 |
| 3.125 | 102. |
| 6.25 | 108. |
| 12.5 | 106. |
| 25 | 107. |
| 50 | 97.8 |

| Standard concentrations(μM) | Betaine Accuracy (%) |
| --- | --- |
| 3.125 | 94.1 |
| 6.25 | 100. |
| 12.5 | 101. |
| 25 | 105. |
| 50 | 105. |
| 100 | 98.5 |

| Standard concentrations(μM) | Butyrobetaine Accuracy (%) |
| --- | --- |
| 0.195 | 101. |
| 0.39 | 103. |
| 0.78 | 94.4 |
| 1.56 | 106. |
| 3.125 | 104. |
| 6.25 | 106. |
| 12.5 | 100. |

| Standard concentrations(μM) | Carnitine Accuracy (%) |
| --- | --- |
| 6.25 | 97.5 |
| 12.5 | 107. |
| 25 | 108. |
| 50 | 107. |
| 100 | 97.7 |
